# Impact of stage, management and recurrence on survival rates in laryngeal cancer

**DOI:** 10.1371/journal.pone.0179371

**Published:** 2017-07-14

**Authors:** Jesper Brandstorp-Boesen, Ragnhild Sørum Falk, Morten Boysen, Kjell Brøndbo

**Affiliations:** 1 University of Oslo, Faculty of Medicine, Institute of Clinical Medicine, Oslo, Norway; 2 Department of Otorhinolaryngology, Division of Surgery and Clinical Neuroscience, Oslo University Hospital, Oslo, Norway; 3 Oslo Centre for Biostatistics and Epidemiology, Research Support Service, Oslo University Hospital, Oslo, Norway; University of South Alabama Mitchell Cancer Institute, UNITED STATES

## Abstract

A retrospective, longitudinal study of 1,616 patients with primary laryngeal squamous cell carcinoma (LSCC) at a single center in Norway during 1983–2010 was undertaken to investigate overall survival, disease specific survival, disease-free survival, prognostic factors for overall survival, and impact of recurrence among all-stage laryngeal cancer patients over 15 years' follow-up. The prognostic impact of gender, age, smoking/alcohol, subsite, tumour, node and metastasis staging, period and modality of treatment were evaluated using Kaplan-Meier and Cox proportional hazard analyses. The importance of recurrence on survival was assessed based on case fatality rates. Five-year overall survival was 56.8%, 64.0% and 38.8%, and disease-specific survival was 80.2%, 87% and 61.6%, respectively, for the entire cohort and for glottic and supraglottic LSCC. Old age, advanced-stage LSCC and supraglottic cancer were associated with lower overall survival. The risk of disease-specific death plateaued after five years and varied significantly by subsite. Multivariate analysis of glottic LSCC revealed that surgical treatment improved overall survival, whereas old age, alcohol, T3-T4 status, positive N-status and no treatment were associated with worse survival. In supraglottic LSCC, age, alcohol, and positive N-status had a significant impact on overall survival by multivariate analysis. Five-year overall survival and disease-specific survival among patients with recurrent disease were 34% and 52%, respectively. In conclusion, marked difference in overall survival between glottic and supraglottic LSCC underline the importance of subsite-specific survival analysis. T-status and primary surgical management is essential only for glottic LSCC, emphasizing the importance of correct disease classification. Inferior outcomes in supraglottic LSCC are associated with old age, positive N-status, and improved follow-up routines are necessary. Primary tumor control is essential since recurrence impairs survival considerably in all subsites. The potential benefit of a primary surgical approach towards T3 LSCC awaits further investigation.

## Introduction

Despite progress in the management of laryngeal squamous cell carcinoma (LSCC) over the last three decades, recent studies show conflicting results regarding survival. Based on a review of the American National Cancer Data Base (NCDB), survival rates following diagnosis of LSCC in the United States seem to have decreased [1] but remain stable in other countries [2]. LSCC mortality varies widely between different European countries, with most Scandinavian countries having low rates [3,4]. In Norway, the estimated age-standardized mortality rate across patients of all ages and both sexes in 2012 was 0.4 per 100,000 for laryngeal cancer compared to 0.7 per 100,000 in the United States [5].

It is essential to consider the highly heterogeneous nature of laryngeal cancer when performing a survival analysis of LSCC. The fact that glottic tumors are diagnosed at an earlier stage compared to supraglottic tumors affects treatment opportunities and prognosis, and argues for survival analysis to be performed according to tumor stage and subsite [6–8]. In Norway, women constitute an increasing proportion of LSCC cases, but LSCC still has the highest male:female ratio of any head or neck neoplasm [9,10]. Previous studies, including analyses in Norway, have found females to be more prone to supraglottic cancer [9,11]. In a recent publication, young age, intermediate T-status and supraglottic cancer were found to be negatively associated with the risk of recurrence, which is expected to ultimately affect outcome [12].

The aim of this study was to analyze the subsite-specific overall survival (OS) among all-stage LSCC patients and to identify prognostic factors for OS. The study also aimed to explore disease-specific survival (DSS) and disease-free survival (DFS) in this cohort, and to examine OS and DSS in patients with recurrent LSCC.

## Materials and methods

All patients treated for LSCC at the Department of Otorhinolaryngology Head and Neck Surgery, Rikshospitalet from 1983 to 2010 were analyzed retrospectively. Patients who had been cured of previous non-laryngeal head and neck cancer, those with concomitant non-laryngeal cancers at the time of LSCC diagnosis were included; patients with previous laryngeal cancer were excluded. Inclusion and exclusion criteria have been described in detail elsewhere [9,12].

Oslo University Hospital, Rikshospitalet and The Norwegian Radium Hospital, are two tertiary academic referral centers which together treat approximately 60% of all patients with laryngeal cancer in Norway. All patients are evaluated by a multidisciplinary team of maxillofacial and head and neck surgeons, radiologists, pathologists and oncologists. Throughout the study period all patients received a uniform and standardized clinical investigation, TNM classification, treatment and follow-up. In the current analysis, follow-up started at the end of treatment and all patients had their first clinical and radiological evaluation 4–6 weeks after the primary treatment. During the first year of follow-up, patients were seen every 8–12 weeks, then 2–3 times during the second and third years. Clinical and histological verification of persistent LSCC within three months of primary therapy was defined as residual tumor, whereas LSCC identified later than three months after the initial treatment in complete responders was denoted as recurrent disease. In cases of residual tumor or recurrence, the date of histological verification was registered and the patient was re-assessed by the multidisciplinary tumor board to decide the appropriate salvage treatment.

Information on follow-up and vital status was obtained through review of medical records and reports from referring institutions. Using a unique personal identification number assigned to the residents of Norway, all individuals diagnosed with cancer are registered in the Cancer Registry of Norway, from which information on national cancer statistics is available [10]. Information regarding the date of death was mainly acquired from a hospital register, which is linked to the National Registry of Death. To obtain an accurate cause of death, the death certificate or autopsy findings were collected. Death was considered to be related to LSCC cancer when laryngeal cancer was documented as the underlying cause of death. Patients who died during treatment, or within three weeks after completion of treatment, were also considered to have died from LSCC, regardless of the actual cause.

The Privacy and Data Protection Office of the CEO Executive Staff at Oslo University Hospital approved the study. Data collection was authorized by the Norwegian Data Protection Authority. Written consent was collected at diagnosis and no patients were lost to follow-up.

### Prognostic factors

Gender, age (≤59, 60–69 and ≥70 years) and information on smoking and alcohol (never, ever or unknown) were documented for each patient at diagnosis. Tumor location was separated into one of three subsites (glottic, supraglottic and subglottic) and the status of tumor, nodal and distant metastasis were classified in accordance with the Union for International Cancer Control TNM classification system (3^rd^ to 6^th^ edition) and the American Joint Committee on Cancer TNM staging (2^nd^ to 7^th^ edition). Early-stage LSCC (Stage I+II) was defined as T1–T2N0M0, and advanced-stage LSCC (Stage III+IV) as T3–T4a/b and any TN+, M+. Moreover, to elaborate on T1a glottic LSCC and the possible impact of treatment, we divided early stage glottic LSCC into T1aN0 and T1b-T2N0. The type of primary treatment for each patient was determined by a collaborative tumor board of head and neck surgeons and oncologists. Accordingly, the primary treatment modalities were categorized as radiotherapy, transoral lasermicrosurgery (TLM), total laryngectomy (TLAR), chemoradiotherapy and palliation/no treatment. The date of treatment and the length of follow-up was categorized as one of four seven-year periods (1983–1989, 1990–1996, 1997–2003 and 2004–2010) and five time intervals (<1 year, ≥1 to <3 years, ≥3 to <6 years, ≥6 to <10 years, and 10–15 years). The management of early and advanced LSCC was divided into non-surgical (radiotherapy, chemoradiotherapy) and surgical (TLM, TLAR) therapy, and OS was compared by treatment for each subsite. Recurrences were categorized as local, regional/loco-regional or distant.

### Statistics

Descriptive statistics were calculated for patient and tumor characteristics and are presented as frequencies and proportions. The three outcomes of interest were all-cause death (OS), disease-specific survival (DSS) and disease-free survival (DFS i.e. no recurrence or death) among LSCC patients.

For OS, DSS and DFS the patients were followed longitudinally from the date of primary treatment to the date of all-cause death, the date of disease-specific death or the date of recurrence, respectively. Follow-up was completed on 30 September 2011 and patients were censored accordingly. Survival rates are presented as Kaplan-Meier plots and five-year observed survival rates. Subsite-specific survival curves for early and advanced-stage LSCC managed by non-surgical and surgical treatment are presented.

Cox regression models were used to evaluate the impact of prognostic factors on OS. Both univariate and multivariate analyses were performed separately for each of the three subsites. Only glottic and supraglottic cancer met the criteria for analysis, in terms of number of patients, and results are presented for these subsites. Risk estimates are presented as hazard ratios (HR) with 95% confidence intervals (CI).

Stacked cumulative incidence curves are presented to illustrate the difference in laryngeal cancer deaths and death from other causes according to subsite over 15 years of follow-up. Areas between the curves are attributed to the probability of disease-specific deaths, deaths from other causes and event-free survival after primary treatment of LSCC.

To estimate case fatality, patients with recurrent LSCC were followed from the date of recurrence to the date of death or end of follow-up on 30 September 2011. Both five-year all-cause death (OS) and DSS among patients with recurrent disease are presented by subsite.

P-values ≤0.05 were regarded as significant. Data analysis was performed using Stata [13] and SPSS [14].

## Results

A total of 1,616 patients were treated for primary LSCC during 1983–2010. A description of the trends in incidence, and risk factors for recurrent LSCC, in the study population has been published previously [9,12].

Of 1,616 patients, 1,126 died (mean age 67.6, range 14–93 years) and 490 were still living (mean age 61.5, range 27–88 years) at the end of follow-up. Primary surgical treatment was administered in 34.5% of early-stage LSCC cases and 27.2% of advanced-stage LSCC cases, respectively. Table 1 summarizes patient and disease characteristics of the study cohort according to survival at the end of 15 years’ follow-up.

**Table 1:** Patient and disease characteristics according to survival at the end of 15 years' follow-up in patients with laryngeal squamous cell carcinoma.

	Death		Alive		Total	
	(n = 1,043 [64.5%])		(n = 573 [35.5%])		(n = 1616)	
	n	%	n	%	n	%
Gender						
Male	917	87.9	487	85.0	1404	86.9
Female	126	12.1	86	15.0	212	13.1
Age (years)						
0–59	202	19.4	256	45.7	458	28.3
60–69	350	33.5	192	33.5	542	33.6
70+	491	47.1	125	21.8	616	38.1
Smoking history						
Never	55	5.3	42	7.3	97	6.0
Ever	924	88.6	511	89.2	1435	88.8
Unknown	64	6.1	20	3.5	84	5.2
Alcohol						
Never	597	57.2	332	57.9	929	57.5
Ever	171	16.4	52	9.1	223	13.8
Unknown	275	26.4	189	33.0	464	28.7
Subsite						
Glottic	678	65.0	449	78.4	1127	69.7
Supraglottic	332	31.8	106	18.5	438	27.1
Subglottic	33	3.2	18	3.1	51	3.2
T-status						
T1	348	33.4	319	55.7	667	41.3
T2	252	24.1	135	23.5	387	23.9
T3	172	16.5	59	10.3	231	14.3
T4	271	26.0	60	10.5	331	20.5
N-status						
N0	814	78.0	530	92.5	1344	83.2
N1	80	7.7	19	3.3	99	6.1
≥N2	149	14.3	24	4.2	173	10.7
M-status						
M0	1,032	99.0	573	100	1602	99.0
M1	11	1.0	0	0	14	1.0
Stage I–IV						
I	335	32.1	316	55.2	651	40.3
II	211	20.2	121	21.1	332	20.5
III	161	15.5	61	10.6	222	13.7
IV	336	32.2	75	13.1	411	25.5
Treatment modality						
Radiotherapy	714	68.5	294	51.3	1008	62.4
Transoral laser microsurgery	115	11.0	217	37.9	332	20.5
Total laryngectomy	135	13.0	44	7.7	179	11.1
Chemoradiotherapy	37	3.5	18	3.1	55	3.4
Palliation/no treatment	42	4.0	0	0	42	2.6
Period of treatment						
1983–1989	318	30.5	85	14.8	403	24.9
1990–1996	279	26.7	98	17.1	377	23.3
1997–2003	272	26.1	130	22.7	402	24.9
2004–2010	174	16.7	260	45.4	434	26.9
Length of follow-up						
<1 year	248	23.8	9	1.6	257	16.0
≥1 to <3 years	274	26.3	92	16.0	366	22.6
≥3 to <6 years	218	20.9	106	18.5	324	20.0
≥6 to <10 years	185	17.7	114	19.9	299	18.5
10–15 years	118	11.3	252	44.0	370	22.9

**Fig 1:**
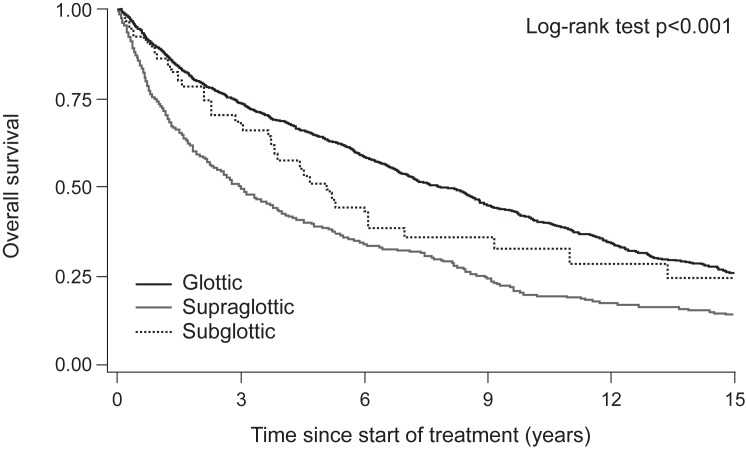
Overall survival of 1,616 LSCC patients according to subsite during 15 years' follow-up.

Five-year OS for the whole cohort was 56.8%, ranging from 38.8% to 64.0% depending on subsite and from 37.4% to 71.0% depending on T-status (Table 2). The median OS was 7.9, 5.1 and 3.0 years for glottic, subglottic and supraglottic LSCC, respectively (Fig 1). Five-year DSS ranged from 61.6% to 87.0%, and five-year DFS from 32.0% to 54.5%, respectively, depending on subsite (Table 2). When analyzed according to T-status, DSS ranged from 47.6% (T4 supraglottic LSCC) to 98.1% (T1a glottic LSCC), and DFS ranged from 23.0% (T3 glottic LSCC) to 69.1% (T1a glottic LSCC), respectively.

**Table 2:** Five-year overall survival (OS), disease specific survival (DSS) and disease-free survival (DFS) according to subsite and T-status among 1,616 patients with laryngeal squamous cell carcinoma.

	Overall			Glottic			Supraglottic			Subglottic		
	%	95% CI		%	95% CI		%	95% CI		%	95% CI	
OS	56.8	54.2	59.2	64.0	61.0	66.8	38.8	34.0	43.5	51.2	36.5	64.2
T1	71.0	67.2	74.4	-		-	40.0	27.7	52.0	-	-	-
T1a	-	-	-	75.7	71.7	79.2	-	-	-	-	-	-
T1b	-	-	-	68.4	51.7	80.3	-	-	-	-	-	-
T2	59.2	54.0	64.0	64.5	58.1	70.2	47.0	37.5	55.9	-	-	-
T3	39.2	32.6	45.8	36.7	28.3	45.1	42.9	31.8	53.5	-	-	-
T4	37.4	32.2	42.7	43.0	35.0	50.9	30.3	23.4	37.5	-	-	-
DSS	80.2	78.0	82.2	87.0	84.8	89.0	61.6	56.3	66.4	81.2	66.8	89.8
T1	94.9	92.7	96.4	-	-	-	69.5	54.0	80.7	-	-	-
T1a	-	-	-	98.1	96.3	99.0	-	-	-	-	-	-
T1b	-	-	-	90.1	75.6	96.2	-	-	-	-	-	-
T2	84.5	80.0	88.1	87.0	81.6	91.0	77.8	67.6	85.1	-	-	-
T3	65.5	58.1	71.9	67.9	58.1	75.8	60.5	47.7	71.0	-	-	-
T4	54.3	48.5	59.8	59.9	51.0	67.8	47.6	39.3	55.4	-	-	-
DFS	48.1	45.5	50.5	54.5	51.4	57.4	32.0	27.6	36.6	43.9	29.9	57.0
T1	64.3	60.3	67.9	-	-	-	37.3	25.3	49.3	-	-	-
T1a	-	-	-	69.1	64.9	72.9	-	-	-	-	-	-
T1b	-	-	-	52.5	36.4	66.3	-	-	-	-	-	-
T2	44.6	39.4	49.6	47.2	40.8	55.3	38.4	29.3	47.4	-	-	-
T3	24.3	18.7	30.3	23.0	15.9	30.8	25.4	16.4	35.3	-	-	-
T4	35.7	31.0	41.0	41.1	33.0	49.0	28.6	21.8	35.7	-	-	-

**Fig 2:**
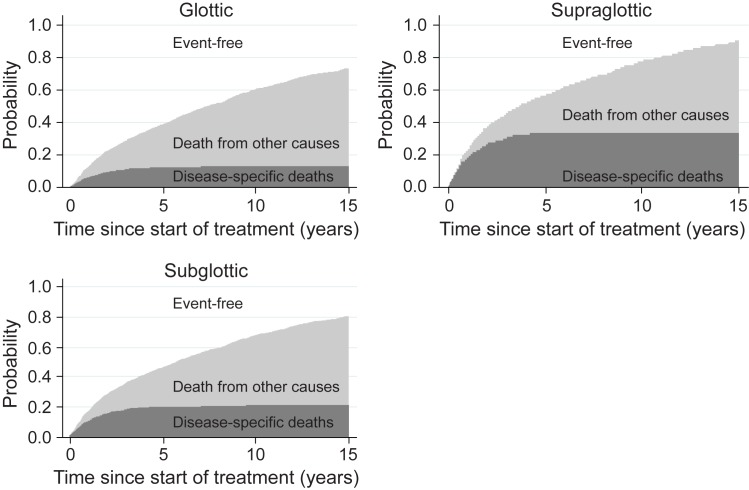
Stacked cumulative incidence of disease-specific death, death from other causes and disease-free survival by subsite during 15 years' follow-up.

Fig 2 presents stack cumulative incidence plots for death due to LSCC or due to other causes according to subsite over 15 years’ follow-up. The occurrence of disease-specific death plateaus at approximately five years, with death from supraglottic, subglottic and glottic LSCC in 38%, 20% and 17% of patients, respectively.

**Fig 3:**
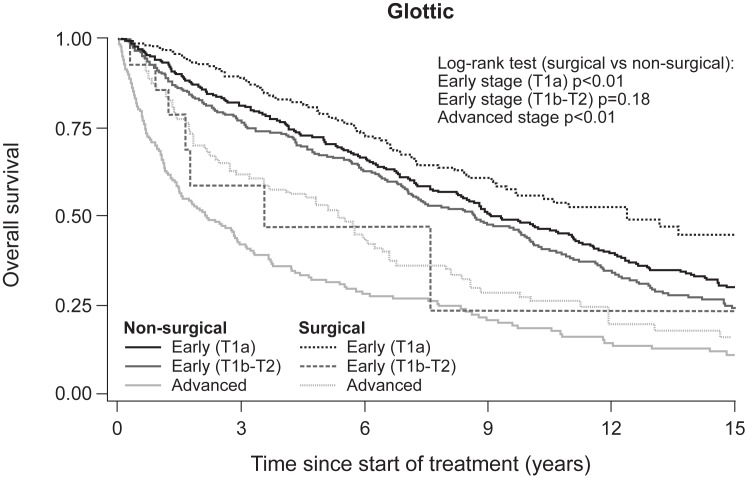
Overall survival of early-stage and advanced-stage glottic LSCC according to non-surgical and surgical treatment.

**Fig 4:**
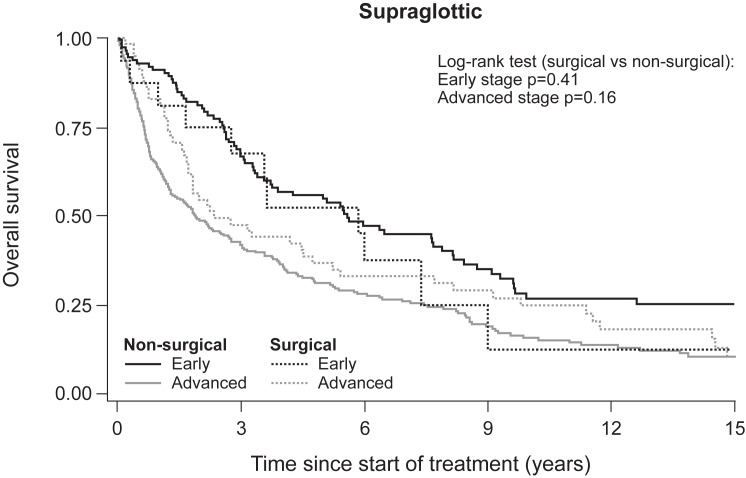
Overall survival of early-stage and advanced-stage supraglottic LSCC according to non-surgical and surgical treatment.

Fig 3 illustrates the difference in five-year OS according to stage and treatment modality in glottic LSCC. Survival was significantly higher in patients treated surgically versus non-surgically in early-stage LSCC (T1a) (log-rank, p = 0.009) and advanced-stage LSCC (log-rank, p = 0.004). However, survival rates among supraglottic LSCC patients showed no significant difference by treatment modality for either early or advanced-stage disease (Fig 4).

Multivariate analysis revealed that age >60 years, use of alcohol, T3–T4 tumors and ≥N2-status were prognostic factors for OS among glottic LSCC patients (Table 3). Of 1,127 patients with glottic cancer, those treated surgically with TLM (HR 0.69, p = 0.04) or TLAR (HR 0.62, p = 0.02) experienced significantly better OS than those treated with radiotherapy or chemoradiotherapy. OS was significantly better for women compared to men with glottic LSCC, but only by univariate analysis (p = 0.046).

**Table 3:** Five-year observed overall survival (OS) and results from Cox regression analysis in patients with glottic LSCC during 15-years' follow-up.

	No. at risk	No. of failures during 15-years	Five-year observed OS			Univariate				Multivariate			
			%	95% CI		HR	95% CI		P value	HR	95% CI		P value
Gender													
Male	1,022	362	62.9	59.8	65.9	1				1			
Female	105	25	75.3	65.7	82.7	0.75	0.56	0.99	0.05	0.84	0.60	1.20	0.34
Age (years)													
0–59	313	63	78.8	73.8	83.1	1				1			
60–69	383	122	66.6	61.5	71.2	2.06	1.64	2.58	<0.001	2.22	1.72	2.86	<0.001
70+	431	202	50.8	45.8	55.7	3.82	3.08	4.75	<0.001	4.36	3.38	5.62	<0.001
Smoking history													
Never	74	24	65.5	52.9	75.5	1				1			
Ever	995	340	64.2	61.1	67.2	1.20	0.86	1.66	0.28	1.38	0.85	2.25	0.19
Unknown	58	23	58.5	44.3	70.2								
Alcohol													
Never	635	205	66.1	62.1	69.7	1				1			
Ever	126	67	46.0	37.0	54.5	1.59	1.27	1.99	<0.001	1.65	1.29	2.10	<0.001
Unknown	366	115	66.7	61.5	71.5								
T-status													
T1a	551	124	75.7	71.7	79.2	1				1			
T1b	44	13	68.4	51.7	80.3	1.15	0.77	1.72	0.49	0.92	0.55	1.53	0.75
T2	248	86	64.5	58.1	70.2	1.44	1.19	1.75	<0.001	1.10	0.83	1.45	0.50
T3	138	82	36.7	28.3	45.1	2.72	2.17	3.41	0.001	2.08	1.51	2.86	<0.001
T4	146	82	42.9	34.7	50.9	2.54	2.03	3.16	<0.001	2.23	1.48	3.38	<0.001
N-status													
N0	1,050	331	66.8	63.8	69.6	1				1			
N1	33	23	30.3	15.9	46.1	2.28	1.55	3.35	<0.001	1.38	0.87	2.21	0.17
N2+	44	33	23.6	12.1	37.2	4.09	2.95	5.68	<0.001	1.66	1.10	2.51	0.01
Stage I-IV													
I	593	136	75.3	71.4	78.7	1							
II	239	79	66.1	59.6	71.8	1.39	1.14	1.68	<0.01				
III	133	78	37.5	28.9	46.1	2.57	2.05	3.23	<0.001				
IV	162	94	40.9	33.1	48.5	2.66	2.16	3.28	<0.001				
Treatment													
Radiotherapy	675	257	61.2	57.4	64.8	1				1			
Transoral laser microsurgery	318	60	78.1	72.6	82.6	0.55	0.45	0.69	<0.001	0.69	0.48	0.99	0.04
Total laryngectomy	102	47	53.1	42.9	62.4	1.29	1.01	1.64	0.04	0.62	0.42	0.93	0.02
Chemo- radiotherapy	16	7	44.1	15.4	69.8	1.39	0.66	2.94	0.39	1.15	0.51	2.61	0.74
Palliation/ no treatment	16	16	-	-	-	30.7	18.0	52.2	<0.001	31.5	14.2	69.8	<0.001
Period of treatment													
1983–1989	293	107	63.5	57.7	68.7	1				1			
1990–1996	249	83	66.7	60.4	72.1	0.84	0.69	1.02	0.09	0.95	0.74	1.21	0.68
1997–2003	287	109	62.0	56.1	67.4	0.99	0.81	1.20	0.91	1.16	0.88	1.53	0.28
2004–2010	298	88	63.8	57.0	69.8	0.97	0.76	1.24	0.82	1.26	0.91	1.75	0.16

Among supraglottic LSCC, age >60 years, use of alcohol, ≥N2-status and treatment by chemoradiotherapy were significant prognostic factors for OS (Table 4). Smoking data were inadequate and were therefore not included in the multivariate analysis.

**Table 4:** Five-year observed overall survival (OS) and results from Cox regression analysis of patients with supraglottic LSCC during 15-years' follow-up.

	No. at risk	No. of failures during 15-years	Five-year observed OS			Univariate				Multivariate			
			%	95% CI		HR	95% CI		P value	HR	95% CI		P value
Gender													
Male	341	205	38.5	33.3	43.8	1				1			
Female	97	53	38.6	28.0	49.1	0.98	0.75	1.27	0.86	0.94	0.70	1.27	0.68
Age (years)													
0–59	129	59	52.9	43.7	61.2	1				1			
60–69	142	87	36.4	28.3	44.6	1.58	1.18	2.11	<0.01	1.95	1.40	2.72	<0.001
70+	167	112	29.8	22.8	37.1	2.02	1.53	2.69	<0.001	2.69	1.95	3.71	<0.001
Smoking history													
Never	22	15	29.2	12.0	48.9	1							
Ever	394	224	40.7	35.6	45.7	1.20	0.86	1.66	0.28				
Unknown	22	19	13.6	34.1	30.9								
Alcohol													
Never	272	147	44.1	38.0	50.0	1				1			
Ever	87	51	25.7	16.5	36.0	1.59	1.27	1.99	<0.001	1.50	1.10	2.05	0.01
Unknown	79	50	33.9	23.4	44.7								
T-status													
T1	65	37	40.0	27.7	52.0	1				1			
T2	123	61	47.0	37.5	55.9	0.83	0.56	1.18	0.30	0.87	0.57	1.34	0.53
T3	84	46	42.9	31.8	53.5	0.94	0.64	1.37	0.74	0.83	0.52	1.31	0.42
T4	166	114	30.3	23.4	37.5	1.35	0.98	1.87	0.007	1.14	0.75	1.75	0.53
N-status													
N0	249	119	50.3	43.8	56.5	1				1			
N1	64	40	35.9	24.0	47.8	1.45	1.06	1.98	0.02	1.29	0.90	1.86	0.17
≥N2	125	99	16.1	9.8	23.7	2.69	2.10	3.46	<0.001	2.32	1.70	3.18	<0.001
Stage I–IV													
I	51	26	45.9	31.4	59.3	1							
II	77	29	60.2	48.0	70.5	0.70	0.45	1.08	0.11				
III	81	40	48.5	36.9	59.2	0.89	0.59	1.36	0.61				
IV	229	163	26.3	20.6	32.3	1.66	1.17	2.36	<0.01				
Treatment													
Radiotherapy	301	164	43.5	37.6	49.2	1				1			
Transoral laser microsurgery	13	7	41.0	13.8	66.9	1.22	0.63	2.69	0.55	1.97	0.83	4.66	0.12
Total laryngectomy	61	36	39.9	27.9	51.9	1.04	0.78	1.42	0.79	0.88	0.60	1.28	0.51
Chemo-radiotherapy	39	27	20.3	8.0	36.6	1.55	1.06	2.27	0.03	1.56	1.00	2.43	0.05
Palliation/no treatment	24	24	-	-	-	9.64	6.12	14.9	<0.001	6.44	3.70	11.3	<0.001
Period of treatment													
1983–1989	100	59	41.0	31.3	50.4	1				1			
1990–1996	119	74	37.8	29.2	46.4	1.05	0.79	1.41	0.72	1.03	0.73	1.46	0.85
1997–2003	104	64	38.5	29.2	47.7	1.03	0.76	1.40	0.82	1.20	0.84	1.73	0.31
2004–2010	115	41	38.9	28.5	49.1	1.06	0.75	1.48	0.75	0.96	0.64	1.45	0.85

Recurrence occurred in 369 patients by the end of follow-up. The case fatality rates by subsite are presented in Table 5. Five-year OS and DSS was 34.0% and 52.2%, respectively. Differences in survival was seen according to subsite type of recurrence. Five-year case fatality rates were lower for patients with regional recurrence compared to patients with local recurrence, while patients with distant recurrence did not survive long enough to permit calculation of five-year survival estimates.

**Table 5:** Five-year case fatality rates by subsite among 369 patients with recurrent LSCC.

	No. at risk	No. of deaths	Overall			Glottic			Supraglottic			Subglottic		
			%	95% CI		%	95% CI		%	95% CI		%	95% CI	
**OS**														
Total	369	284	34.0	29.0	38.9	39.5	33.2	45.8	21.3	14.1	29.6	41.7	15.3	66.5
Local	275	200	42.2	36.2	48.1	45.2	38.0	52.1	31.3	20.3	42.8	62.5	22.9	86.1
Regional	63	54	14.0	6.6	24.2	19.5	7.6	35.5	10.0	2.6	23.6	-	-	-
Distant	31	30	-	-	-	-	-	-	-	-	-	-	-	-
**DSS**														
Total	369	161	52.2	46.5	57.5	60.4	53.4	66.7	34.7	25.3	44.2	55.6	23.1	79.0
Local	275	94	62.2	56.0	68.0	67.5	60.0	74.0	46.4	33.2	58.6	72.9	27.6	92.5
Regional	63	44	25.7	14.0	38.0	32.2	15.0	51.0	22.2	9.2	38.7	-	-	-
Distant	31	23	-	-	-	-	-	-	-	-	-	-	-	-

## Discussion

In this unselected cohort of 1,616 LSCC patients, OS at five years was 56.8%. Age, subsite, T-status, N-status, modality of treatment and recurrent disease all exerted a marked impact on OS survival. DSS and DFS were 80.2% and 48.1%, respectively.

Not surprisingly, older age impaired the outcome independent of subsite or treatment. Since the population is aging in most European countries and in North America, this subgroup will likely challenge current treatment guidelines in the future.

The five-year OS and DSS rates for glottic LSCC parallels the findings from a four-decade national population-based study from Denmark, in which 5,132 patients with stage I–IV glottic LSCC were evaluated [15]. Our study shows that older age and advanced T- and N+ status are negatively associated with survival. In the Danish cohort, 96% were treated by primary radiotherapy, and 2% of all tumors were T4. In our unselected group, almost one-third of patients received surgery as primary treatment and 20% of tumors were T4. Thus, the survival rates in the two studies, were based on crucial differences in cohort characteristics and primary therapy. Moreover, although our patients were not randomly assigned to different primary treatments, we found primary surgical intervention to be a positive prognostic factor of OS in both early (TLM) and advanced (TLAR) glottic LSCC. This corresponds with the results of a meta-analysis by Higgins *et al* comparing TLM versus radiotherapy for the treatment of early-stage glottic cancer [16]. The meta-analysis showed no significant difference between TLM and radiotherapy for local control, but for OS the analysis favored TLM. In studies from the American NCDB by Chen *et al*, TLAR was also associated with improved survival compared to non-surgical management of advanced-stage LSCC [17,18]. Prior to the studies based on NCDB, results from the Surveillance, Epidemiology, and End Results (SEER) Program in the United States showed evidence of a decrease in DSS between mid-1980 and 1990 [19], which was subsequently elaborated in a large (158,426 patients) retrospective study by Hoffman and colleagues 1985–2001 [1]. The decrease in survival reported by Hoffman coincided with extended use of non-surgical therapy, whereas the relative survival of T3N0M0 tumors (all sites) seemed to improve when treated with primary surgery or surgery plus radiotherapy compared to radiotherapy or chemoradiotherapy.

Historically, survival of T3 laryngeal cancer has been better than T4 laryngeal cancer [20,21]. However, in the current study, there was no difference in OS among T3 and T4 LSCC overall, or specifically in glottic LSCC. Since most T3 LSCC in our cohort was treated non-surgically by radiotherapy or chemoradiotherapy, and the majority of T4a surgically by TLAR and postoperative radiotherapy (50 Gy), these findings merit attention. Our results are consistent with a study from the Netherlands concerning 10-year outcomes following organ-sparing versus organ-sacrificing management of T3-T4 laryngeal cancer [22]. Timmermans and co-workers found no significant difference in five-year OS between T3 LSCC (primarily treated by radiotherapy or chemoradiotherapy) and T4 LSCC (primarily treated by TLAR). In a subsequent study, Timmermans showed similar survival rates for T3 LSCC, regardless of the modality of primary treatment (TLAR, radiotherapy or chemoradiotherapy) [23]. Dziegielewski *et al* suggested a reassessment of current treatment guidelines based on data from The Alberta Cancer Registry showing superior survival in patients treated surgically for T3 and T4a LSCC compared to those treated non-surgically (radiotherapy and chemoradiotherapy) [24]. Similarly, in a recent publication from the SEER database, Megwalu found a 30% higher risk of mortality in patients treated non-surgically versus surgically for stage III and IV LSCC [25].

The direct effect of treatment in observational studies must be interpreted with caution due to the possibility of treatment selection bias. We found no significant difference in five-year OS survival between the four study intervals. Possible explanations could be, that there is no difference, alternatively that the subgroups within the different treatment modalities are too small or that the specific treatment have not been adopted long enough to affect results markedly. However, we found significant effects of treatment over time. The prognostic relevance of non-surgical (organ-preserving) versus surgical management of advanced-stage LSCC has been discussed extensively since the Veterans Affairs Laryngeal Cancer Study [26]. There is a high level of consensus on the use of TLAR in T4a LSCC, but the clinical diversity and heterogeneity of T3 laryngeal cancers represent a major challenge to established guidelines [17,27]. Based on the idea of organ preservation, TLM and open/endoscopic partial laryngectomies have been proposed as therapeutic alternatives to radiotherapy or chemoradiotherapy in several publications [28,29]. Canis *et al* and Peretti and colleagues have reported five-year OS of 64.4% and 63.3% in two studies where TLM was used as the primary approach to T3 glottic and supraglottic LSCC, respectively [30,31]. Both authors highlighted the favorable functional outcome but stressed the importance of careful patient selection. During the last decade, we have applied TLM to selected cases of T2 glottic and supraglottic LSCC but TLM is not yet considered standard procedure, in the case for T3 LSCC at our institution.

In line with previous reports, supraglottic cancer (any T) was associated with distinctly inferior OS and DFS compared to glottic cancer. The exception was for patients presenting with a T3 glottic LSCC, in whom five-year OS was comparable to supraglottic LSCC. However, in our cohort, disease-specific death was twice as frequent among patients with supraglottic LSCC as in patients with glottic LSCC. Several authors have questioned the non-surgical approach towards supraglottic LSCC, which is currently practiced almost worldwide. In our cohort, the glottic:supraglottic ratio in early-stage LSCC was 6.5:1, whereas the ratio was 1:1 in advanced-stage LSCC. Multivariate analysis in supraglottic LSCC revealed that older age (>60 years), N2+ status and chemoradiotherapy were significant negative prognostic factors for OS, whereas T-status had no significant impact on outcomes. The negative impact of age and N+ status is consistent with the findings from Ganly *et al* [32], who investigated 182 cases of advanced-stage supraglottic LSCC treated at the Memorial Sloane-Kettering Cancer center. Contrary to the findings of Ganly *et al*, chemoradiotherapy showed a borderline negative associaton with OS in our cohort, whereas the remaining treatment modalities had no significant impact on the outcome. Since chemoradiotherapy has been applied only since 2000 at our institution, the findings must be interpreted with caution. In a retrospective study of 653 supraglottic LSCC patients by Sessions *et al*, the survival rates of nine different treatment modalities (mainly surgical) were compared [33]. Results showed that older age (>65 years), N+-status, advanced (T3-T4) disease, non-free resection margins and recurrence were key predictors of DSS. The authors highlighted subtotal supraglottic laryngectomy because of the high laryngeal preservation rate and focused on the importance of follow-up for at least eight years. The idea of primary surgical intervention in supraglottic LSCC is supported by a study of Harris *et al* in 6797 patients from the SEER database, where 928 patients with supraglottic LSCC underwent primary surgery (mostly laryngectomy) [34] and in which a significant OS and DSS benefit was found. Our management of supraglottic LSCC was in accordance with the Danish Head and Neck Cancer Study guidelines [35]. More than 80% of early-stage supraglottic LSCC were managed by radiotherapy, while 75% of advanced-stage tumors were treated by radiotherapy or chemoradiotherapy, with or without complementary neck dissection. Among patients with advanced supraglottic LSCC managed surgically at our institution, TLAR with or without neck dissection was the typical approach. Although we cannot present the survival benefit according to surgical treatment modalities of supraglottic carcinomas in our cohort, we agree that this issue warrants attention [36]. Further studies are required to substantiate the possible advantages in OS and DSS of primary surgery, not only in advanced supraglottic LSCC but also in advanced (non-T4a) glottic LSCC.

Patients with recurrent disease had lower five-year OS (34.0%) than in the cohort as a whole (56.8%). When comparing prognostic factors for OS in the current study with the risk factors for recurrence in the same cohort published previously, old age was a positive factor with regard to the risk of recurrence, and negatively affected OS [12]. T-status was a significant prognostic factor for both OS (T3-T4) and the risk of recurrence (T1b-T4) in glottic LSCC, but the primary treatment only had an impact for OS. Supraglottic LSCC *per se* was a negative prognostic factor with regard to the risk of recurrence and for OS. In order to reduce the risk of subsequent death, patients at risk of recurrence should be observed closely as recurrent LSCC, and the localization of recurrent LSCC (local, regional, distant), has a significant impact on results.

The strengths of this study included the size and unselected nature of the LSCC cohort. The accuracy of data from the date of diagnosis until the last follow-up, censoring or death is enhanced by its single-center nature. Potential shortcomings may be the retrospective nature of the database, which makes it impossible to control all variables or to exclude biases and confounders. The short follow-up time of the patients who were enrolled most recently, and a general risk of under-reporting of patients managed outside our institution during follow-up, must also be considered. However, given the standardized clinical regimen practiced at our center during diagnosis, treatment and follow-up, as well as the well-established cooperation with the referring otorhinolaryngological departments, we have no indication of such biases. The use of different treatments in early and advanced-stage LSCC provide possible therapeutic confounders as well as treatment selection bias. Also, the concurrent development in diagnostic and post-treatment imaging surveillance might have affected tumor classification and the ability to define recurrent cases accordingly. However, in our effort to present the adjusted hazard ratios of multiple risk factors as they were presented in everyday clinical practice, we considered multivariate Cox regression the best possible method for analysis. Moreover, to illustrate the marked differences in outcome based on tumor localization, we presented a subsite-specific survival analysis.

## Conclusions

This study emphasizes the importance of subsite-specific laryngeal cancer survival analysis since both survival rates and the impact of prognostic factors vary by LSCC subsite. Early-stage glottic LSCC has excellent survival rates. Old age, advanced-stage LSCC and supraglottic carcinomas were associated with an unfavorable outcome. The unfavorable impact of supraglottic LSCC, regardless of initial T-status, calls for close follow-up. Recurrence reduces survival considerably in all subsites and highlights the need for primary tumor control. More studies are required to evaluate the potential gains associated with primary surgical treatment of advanced LSCC in general, and whether follow-up of supraglottic LSCC should be intensified.
